# Evaluation of Pirfenidone for the Treatment of Acute Respiratory Distress Syndrome: A Case Report

**DOI:** 10.4236/jbm.2026.143008

**Published:** 2026-03-03

**Authors:** Carlie Cressey Brown, Shelby Evans Pons, Megan Anne Watson, Tyree Heath Kiser, James Charles Lavelle

**Affiliations:** 1 Department of Clinical Pharmacy, University of Colorado Skaggs School of Pharmacy and Pharmaceutical Sciences, Aurora, CO, USA; 2 Department of Medicine, University of Colorado School of Medicine, Aurora, CO, USA

**Keywords:** Acute Respiratory Distress Syndrome, ARDS, Pirfenidone

## Abstract

**Background:**

Acute respiratory distress syndrome (ARDS) is an aggressive, inflammatory lung injury with a high mortality rate. While accepted management includes lung protective ventilation strategies, there is currently no mainstay pharmacologic treatment for ARDS. Adjunctive pharmacologic treatment may include glucocorticoids, neuromuscular blockade and inhaled pulmonary vasodilators. Due to its anti-inflammatory, antioxidant, and anti-fibrotic properties, pirfenidone presents as a potential therapeutic option for patients with ARDS.

**Case Report:**

We present a patient treated with pirfenidone for ARDS. Our patient was a 31-year-old man who presented to the hospital with dyspnea on exertion and concern for relapsed acute myeloid leukemia. After a complex hospital course, the off-label use of pirfenidone 801 mg three times daily was pursued to treat his ARDS. The patient’s ARDS resolved after 10 days of pirfenidone, with no adverse effects, and he was discharged.

**Conclusions:**

This case illustrates the potential utility of pirfenidone in the management of ARDS. After no improvement with widely accepted strategies including lung protective ventilation and steroids, the patient demonstrated recovery after the initiation of pirfenidone. We can infer correlation but not causation in this setting, prompting the need for further prospective randomized clinical trials to establish pirfenidone as a therapeutic option in ARDS.

## 1. Background

Acute respiratory distress syndrome (ARDS) is an acute hypoxemic lung injury typically caused by a predisposing factor such as infection, aspiration, transfusion or shock. ARDS has historically been defined using the Berlin 2012 criteria which requires the 1) acute onset (within 1 week) of 2) non-cardiogenic pulmonary edema with 3) bilateral opacities on chest radiography and 4) PaO_2_:FiO_2_ ratio <300 mmHg among patients who are intubated. The updated 2023 American Thoracic Society (ATS) definition expands to include patients who are not intubated, incorporates both PaO_2_:FiO_2_ or SpO_2_:FiO_2_ measurements and allows ultrasound as an acceptable imaging modality, particularly in resource-limited settings [1] [2]. ARDS progresses through four stages, which include exudative, proliferative, fibrotic, and recovery stages. ARDS is a major cause of intensive care unit (ICU) admission with about 10% of ICU patients and 23% of mechanically ventilated patients meeting the criteria for ARDS diagnosis in a 2016 study [3] [4]. Despite this high incidence, ARDS is very heterogenic in its causes, manifestations, and treatment [3]. Both direct and indirect insults can cause ARDS but sepsis, mainly from pneumonia, is the most common direct cause of ARDS in adult patients [5].

With no mainstay pharmacologic treatment, mortality rates remain high at 35% to 45% [5]. Treatment is generally focused on minimizing inflammation and reversing the underlying etiology while supportive care is utilized to prevent further complications. Lung protective ventilation strategies and prone positioning are recommended for all patients with moderate to severe ARDS to improve oxygenation and potentially reduce mortality [6]-[8]. Pharmacologic treatment options include glucocorticoids, neuromuscular blockers, and inhaled pulmonary vasodilators [3] [7] [9]. Here we present a patient with ARDS secondary to pneumonia who was refractory to standard therapies and effectively treated with pirfenidone.

## 2. Case Report

Our patient was a 31-year-old male with medical history of acute myeloid leukemia (AML) and dual cord stem cell transplantation three years prior to admission. The patient presented to the hospital with bleeding gums when flossing, dyspnea on exertion, headache, and concern for relapsed AML. On hospital day 13, the patient experienced an increased work of breathing requiring 3 to 5 L/min nasal cannula. Despite this intervention, the patient’s respiratory status continued to decline, with an O_2_ saturation of 87% while on 5 L/min nasal cannula. On hospital day 17, the patient was transferred to the ICU and intubated due to worsening acute hypoxic respiratory failure and shock. Soon after intubation, he was started on lung protective ventilatory settings and placed in the prone position. The patient remained intubated for 18 days during which there was an ongoing concern for infection. His FiO_2_ and PEEP requirements slowly declined from 60% to 40% and 18 to 8 cmH_2_O, respectively. After passing spontaneous awakening and breathing trials, he was extubated on day 34, following resolution of his acute respiratory failure. Unfortunately, the patient rapidly declined while on heated high flow nasal cannula, with labored and shallow work of breathing and tachypnea. The following day, he required re-intubation and plans were made for a future tracheostomy.

**Figure 1.**
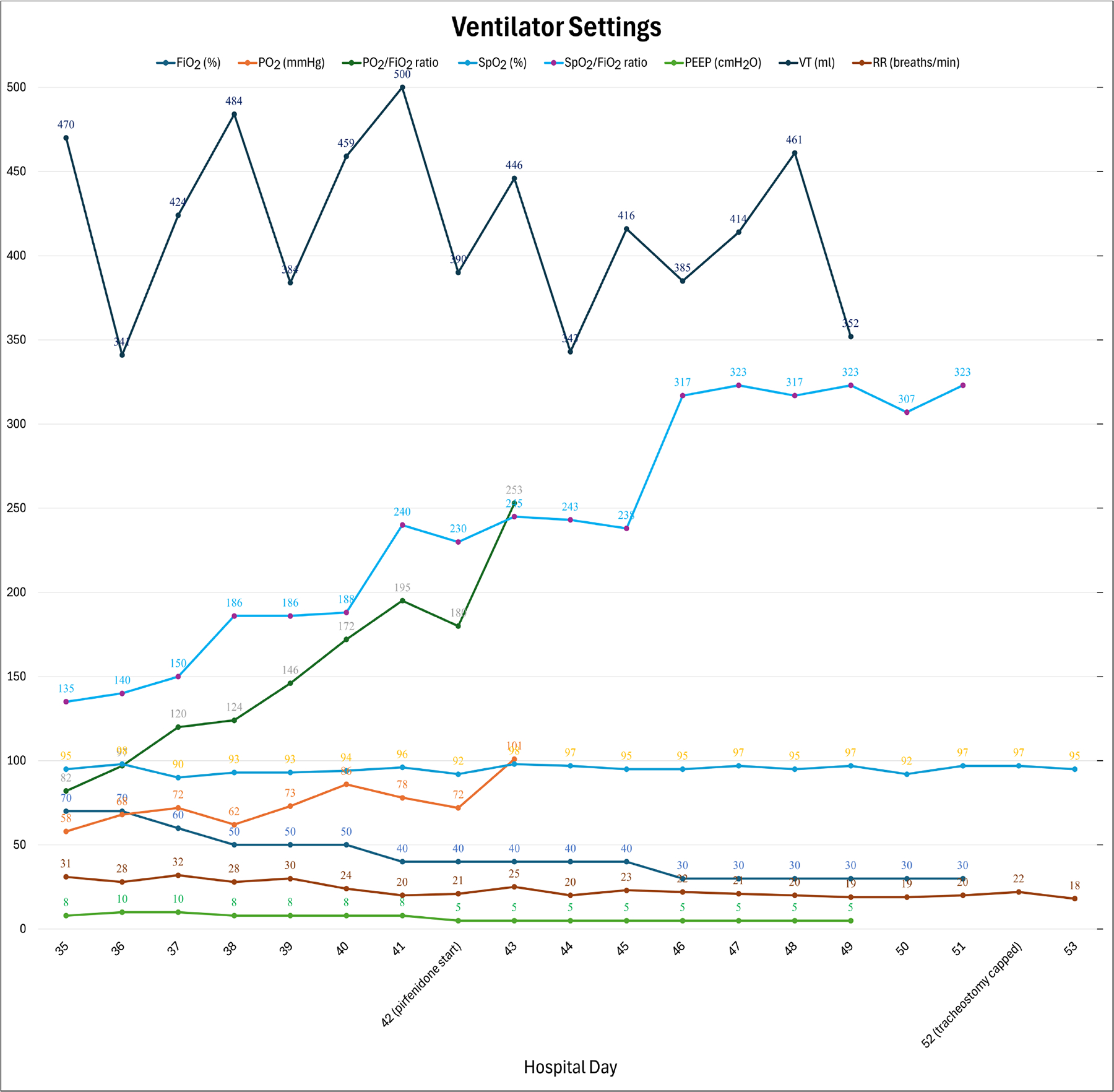
Ventilator Settings. Data depicts the clinical course 7 days prior and 11 days following the initiation of pirfenidone therapy. FiO_2_: fraction of inspired oxygen. PO_2_: partial pressure of oxygen. P/F ratio: ratio of partial pressure of oxygen (P) to the fraction of inspired oxygen (F). SpO_2_: oxygen saturation. SpO_2_/FiO_2_ ratio: ratio of oxygen saturation to the fraction of inspired oxygen. PEEP: positive end-expiratory pressure. VT: tidal volume. RR: respiratory rate.

**Figure 2.**
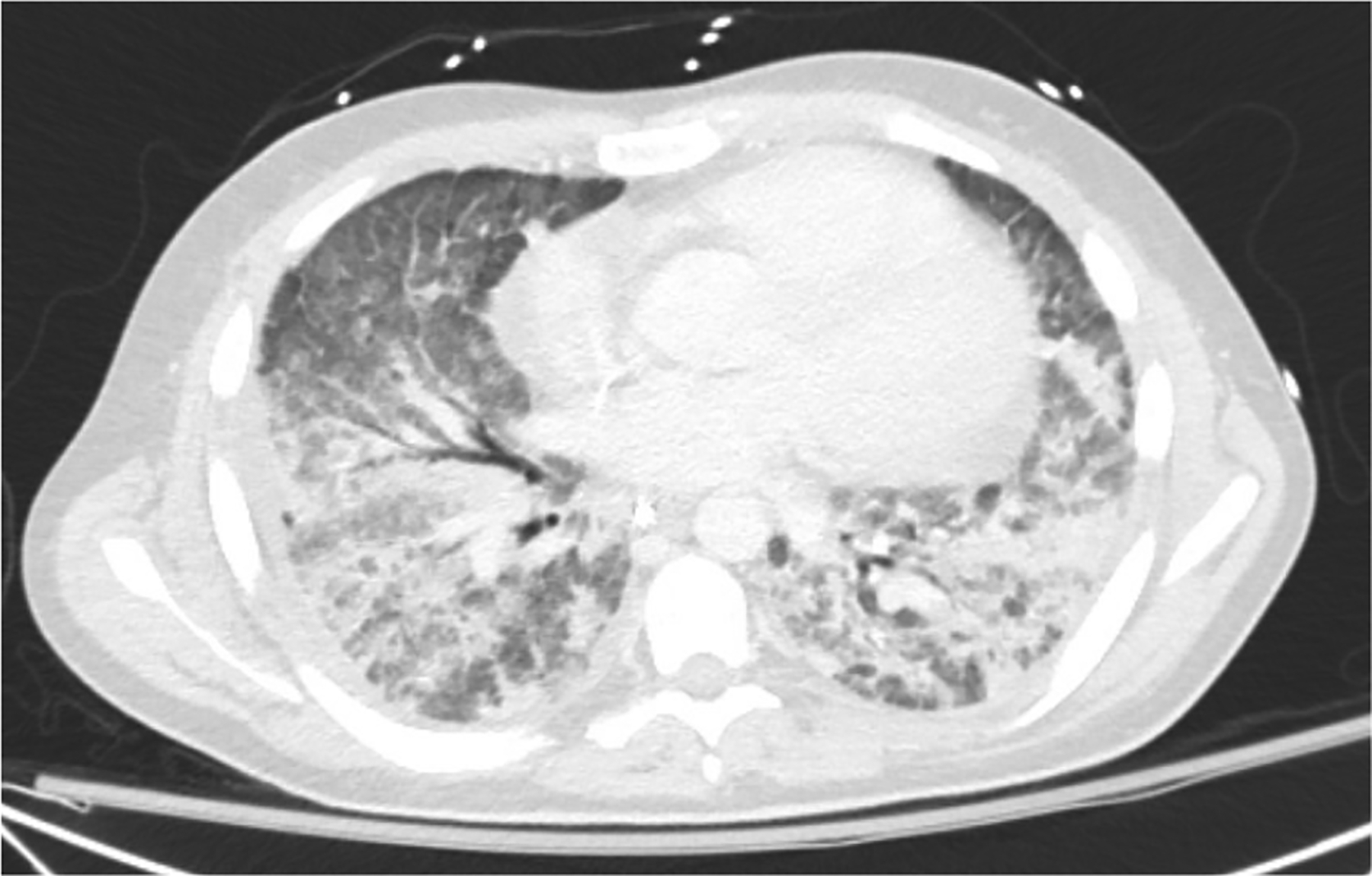
Axial computed tomography (CT) image, corresponding with hospital day 35 in Figure 1, demonstrating extensive bilateral airspace consolidation and areas of ground glass attenuation.

**Figure 3.**
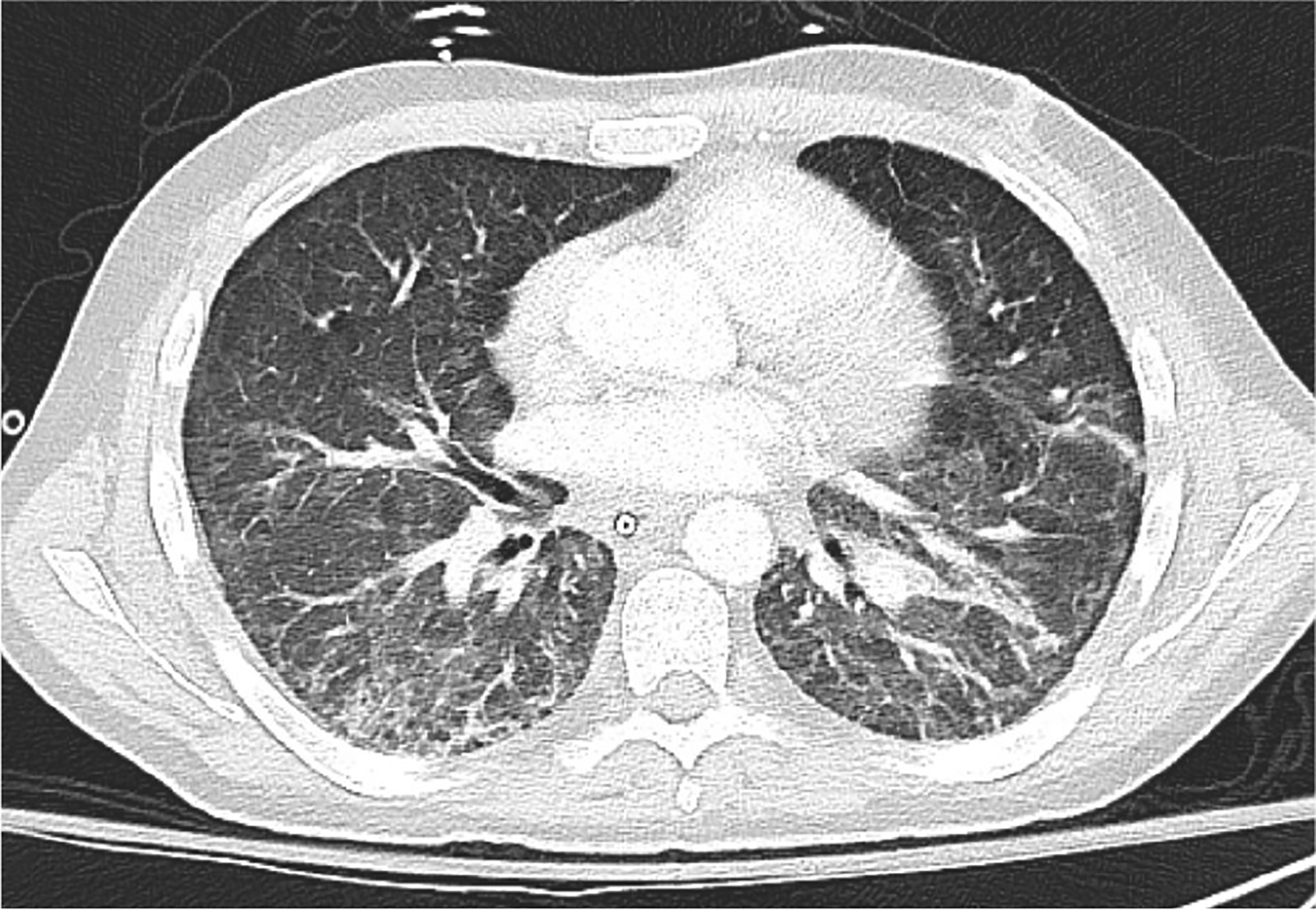
Axial CT image, corresponding with hospital day 49 in Figure 1. Compared to Figure 2, there is significant improvement of the patchy bilateral airspace consolidation. There remains diffuse, centrilobular ground glass opacities on a background of interlobular septal thickening with minimal consolidative changes in the lung bases. There are no new areas of consolidation.

**Figure 4.**
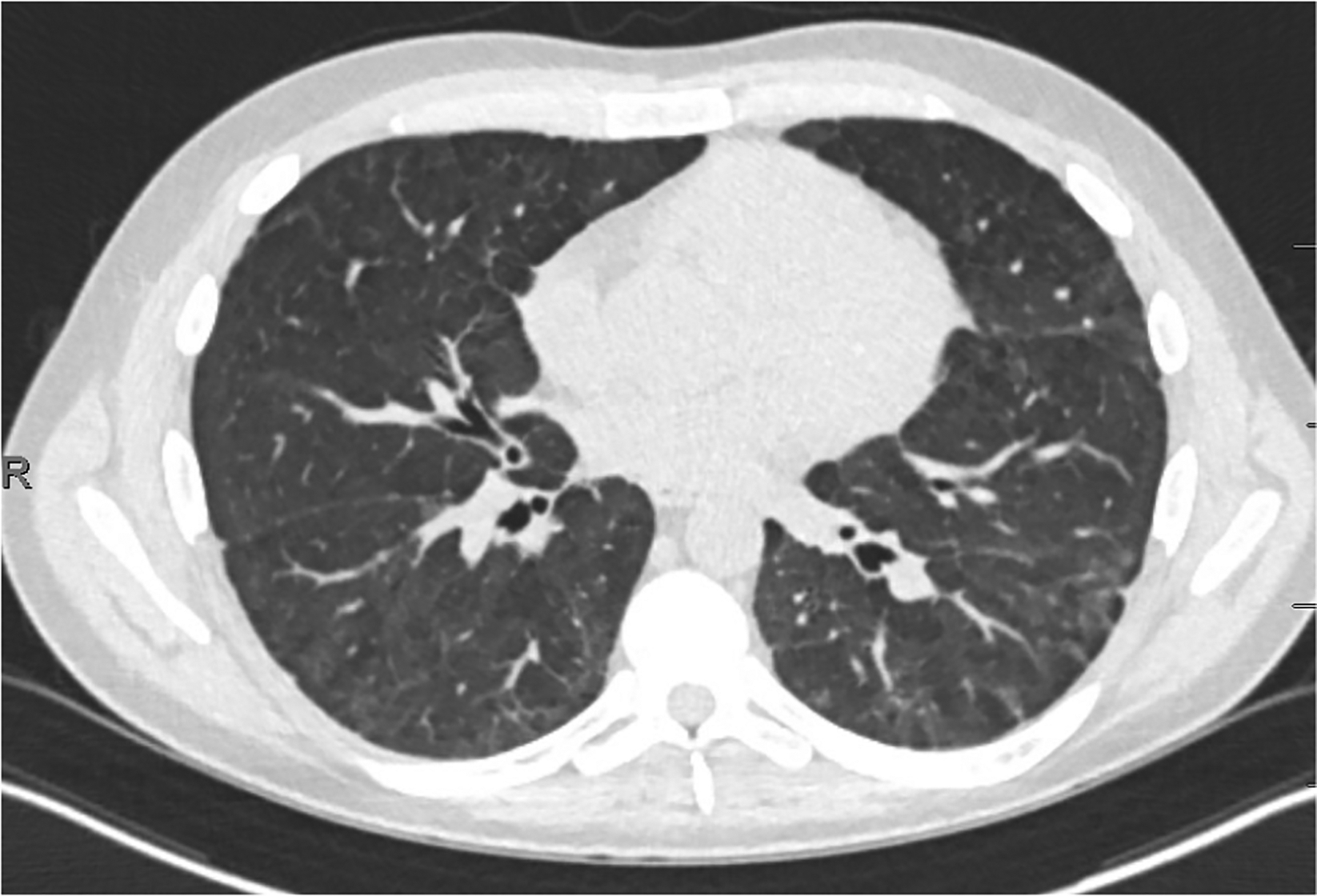
Axial CT image corresponding to 30 days post discharge and one week post completion of pirfenidone therapy. Continued improvement in centrilobular ground glass opacities with interval resolution of consolidative components and improving interlobular septal thickening. Evolving subpleural linear and bandlike opacities, greatest in the lingula and posterior lung bases.

Upon re-intubation, bronchoalveolar lavage was positive for aspergillus galactomannan (Figure 2). A diagnosis of ARDS was confirmed and presumed to be due to fungal pneumonia. Lung protective ventilation was continued with a FiO_2_ of 70%, PEEP of 8 cmH_2_O, and tidal volume of 470 mL (7.4 mL/kg of ideal body weight). Notably, a tidal volume of 7.4 mL/kg of ideal body weight exceeds the guideline target of 6 mL/kg, but tidal volumes ranging between 4 mL/kg and 8 mL/kg were allowed in the intervention arm in the ARMA trial so long as plateau pressures were less than 30 cm H_2_O [10]. However, the patient was kept on an average tidal volume of 6.5 mL/kg for the remainder of the intubation and plateau pressures remained below 30 cm H_2_O. Additional ventilator settings throughout the course of hospitalization are displayed in Figure 1: Ventilator Settings. On hospital day 39, a tracheostomy was placed, and ventilatory support was continued. Dexamethasone 20 mg was initiated on hospital day 40 and scheduled for five days before transitioning to 10 mg for an additional five days following the DEXA-ARDS protocol [11]. Due to high minute ventilation that precluded liberation from mechanical ventilation, which was presumed secondary to fibrotic changes (Fibrotic phenotype was suspected based on persistent bilateral reticulation, traction bronchiectasis, and reduced lung compliance on serial CT imaging), the off-label use of pirfenidone 801 mg three times daily for one month was started on hospital day 42. Prior to the start of pirfenidone, the patient required FiO_2_ of 60%, PEEP of 10 cmH_2_O, and an average tidal volume of 7mL/kg to achieve a PaO_2_:FiO_2_ of 90. One day after the initiation of pirfenidone, blood gases were no longer collected, but improvements are shown utilizing SpO_2_:FiO_2_ ratios as a surrogate marker for PaO_2_:FiO_2_ in Figure 1. Within a few days after initiating pirfenidone, the level of support rapidly decreased to FiO_2_ of 30%, PEEP of 5 cmH_2_O and average tidal volume of 6.3 mL/kg to achieve an average SpO_2_:FiO_2_ of 318. Ventilator settings trends can be viewed in Figure 1. On hospital day 43, he began weaning from the ventilator. By hospital day 47, five days after starting pirfenidone, the patient was tolerating tracheostomy collar during the day and able to rest on the ventilator overnight with minimal ventilatory support. Another CT image was taken on hospital day 49 (Figure 3) demonstrating significant improvement of the patchy bilateral airspace consolidation. Ten days after the initiation of pirfenidone, the tracheostomy tube was capped and the acute hypoxic respiratory failure due to ARDS had resolved. Liver function tests were monitored at least weekly to assess pirfenidone safety and remained within normal limits. No adverse effects were noted related to pirfenidone therapy throughout the duration of treatment. Both antifibrotic therapy (pirfenidone) and prolonged antifungal treatment (posaconazole) were continued after discharge. The 30-day course of pirfenidone was completed and posaconazole was continued for 45 days post discharge. Respiratory improvement without clear resolution of fungal infection, suggesting potential contribution of pirfenidone to the recovery. A final CT image was collected 30 days post discharge and one week post completion of pirfenidone therapy, revealing continued improvement (Figure 4). Pirfenidone treatment duration was guided by the PIONEER protocol (~28 days) and reassessed based on clinical response.

## 3. Discussion

This case demonstrates the potential safe and effective use of pirfenidone as a therapeutic option for patients with ARDS. After an adequate trial of standard ARDS interventions including lung protective ventilation, corticosteroids, and prone positioning, the off-label use of pirfenidone was pursued and was temporally associated with resolution of the patient’s ARDS without any drug-related complications. Although dexamethasone was initiated two days prior to pirfenidone, raising the possibility of delayed corticosteroid effects, the patient had shown minimal sustained improvement before antifibrotic therapy was started. Corticosteroid responses in ARDS are often gradual and variable, whereas this patient demonstrated a more rapid and consistent improvement following pirfenidone initiation. While the temporal overlap limits definitive attribution, the timing and magnitude of clinical recovery suggest that pirfenidone may have played an important contributory role beyond corticosteroid therapy alone.

Pirfenidone is an anti-fibrotic drug with adverse effects that include skin rash, fatigue, and gastrointestinal upset which can be mitigated through a titration period up to the recommended daily dose. The exact mechanism of action is not fully understood, but pirfenidone possesses anti-inflammatory, antioxidant, and antifibrotic properties [12] [13]. Pirfenidone is FDA-approved to treat idiopathic pulmonary fibrosis (IPF), but little data exists regarding its utility in the treatment of ARDS. Despite differences seen between ARDS-related fibrosis and other pulmonary fibrotic processes such as IPF, the foundational mechanisms of pulmonary fibrosis are often shared [14].

Pirfenidone has been under study for decades in a variety of models of lung injury and fibrosis with most of the data limited to animal models [4] [15]. A study performed in 2022 utilized lipopolysaccharides (LPS) to induce ARDS in mice to assess the therapeutic effects of pirfenidone in lung inflammation and injury. Compared to the control group, pirfenidone treatment prolonged survival and reduced lung injury in LPS-induced mice. One major highlight of the study is pirfenidone treatment inhibited the progression of pulmonary fibrosis ultimately reducing the mortality of ARDS mice in the early stages of ARDS, specifically the first 7 days. These findings make pirfenidone a promising agent to reduce ARDS associated pulmonary fibrosis [12]. Furthermore, a recently published review thoroughly analyzed the molecular mechanisms of pirfenidone including its anti-inflammatory capabilities. Pirfenidone’s ability to diminish the production of cytokines, reduce the accumulation of inflammatory cells, prevent inflammasome activation, and limit oxidative stress response makes it an appealing choice to treat inflammation in lung disorders such as COPD, cystic fibrosis, and ARDS [16]. The findings in these studies supported the clinical decision to initiate pirfenidone in our patient [12] [16]. Additionally, the PIONEER study, a large multicenter randomized control trial, estimated to include 130 participants, is currently underway to investigate the efficacy of pirfenidone in ARDS patients. The protocol for this study was used to identify the appropriate dose of pirfenidone, 801 mg three times a day, for the patient treated in our case study [4].

More recently, following the COVID-19 pandemic, a larger pool of data has become available related to the treatment of COVID-19 associated ARDS with pirfenidone. Despite the large range of clinical factors COVID-19 can present with, ARDS is the primary cause of mortality in these patients [17]. SARS-CoV-2 triggers a rise in inflammatory markers which closely resembles the pathophysiological mechanisms of IPF and ARDS. One review highlights pirfenidone’s potential for simulating the recovery phase and controlling the fibroproliferative processes seen in ARDS [17]. The degree of fibroproliferation in ARDS is strongly correlated with poor prognosis, including mortality and ventilator dependence. Consequently, controlling and limiting this process could provide better patient outcomes [14]. A recently published case study, reviewing a patient with post-COVID-19 pulmonary fibrosis treated with pirfenidone, provides further support for the use of pirfenidone in ARDS patients. Initially admitted for COVID-19, the 66-year-old female patient’s hospitalization was prolonged due to the development of a bacterial infection and ARDS. She received broad spectrum antibiotics, invasive mechanical ventilation, and extracorporeal membrane oxygenation (ECMO). Her hypoxemia improved allowing for weaning from ECMO support, but chest CT scans a month later showed diffuse interstitial changes consistent with interstitial lung disease (ILD). Pirfenidone was started at 600 mg per day and escalated to 600 mg three times daily by the end of week one to maintain a total daily of 1800 mg. After 2 months of therapy, the patient’s dyspnea and pulmonary fibrosis improved, allowing her to be discharged. Pirfenidone therapy was continued for two years, and pulmonary function tests showed improvement in restrictive ventilatory impairments and diffusion capacity [13]. From this data, we can infer pirfenidone may be effective in controlling and treating ARDS-associated inflammation and pulmonary fibrosis. Our case report adds to the current body of literature supporting the use of pirfenidone in ARDS.

We report the improvement in ARDS following initiation of pirfenidone. The anti-inflammatory, antioxidant, and antifibrotic properties of pirfenidone may attenuate ARDS related pulmonary fibrosis when other therapies have been trialed with no success. Further prospective clinical trials are needed to establish pirfenidone as a therapeutic option in ARDS.
